# Supplementary material for: A novel CD69 binding ankyrin repeat protein (Ankyron®) enables detection of early activated and antigen-specific T cells in pigs
Source: Sci Rep. 2026 Aug 1;16:35781. doi: 10.1038/s41598-026-64514-8 (PMC13719496; doi:10.1038/s41598-026-64514-8)
Supplement: Supplementary file 1 [file 41598_2026_64514_MOESM1_ESM.pdf]

Supplementary Material

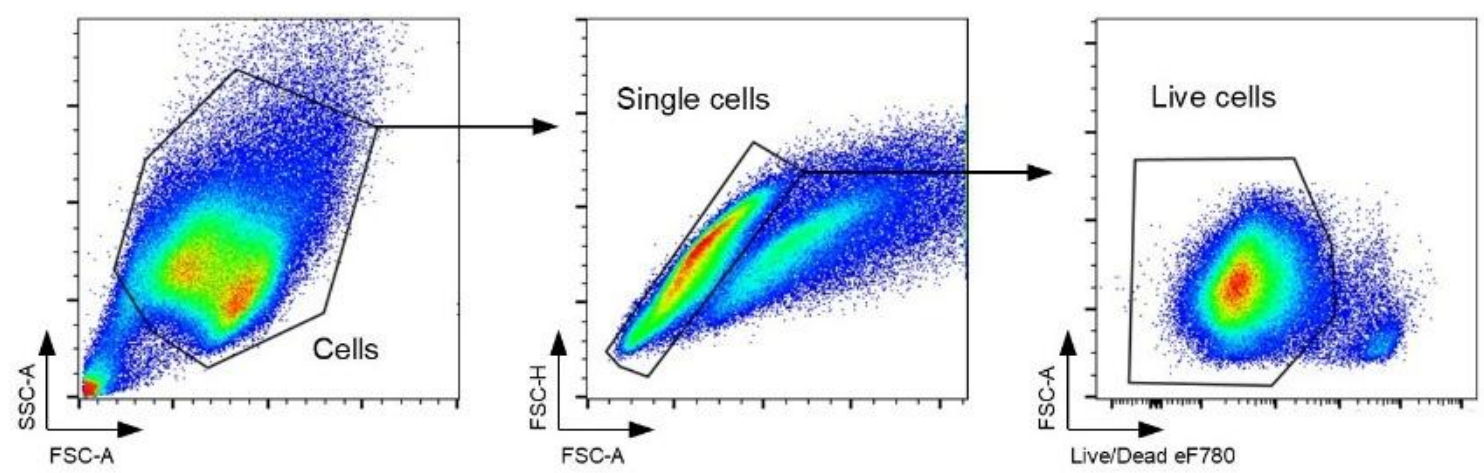

Supplementary Figure 1. Gating strategy for HEK293T cells. Cells were gated according to their light scatter properties, followed by exclusion of doublet cells and dead cells.

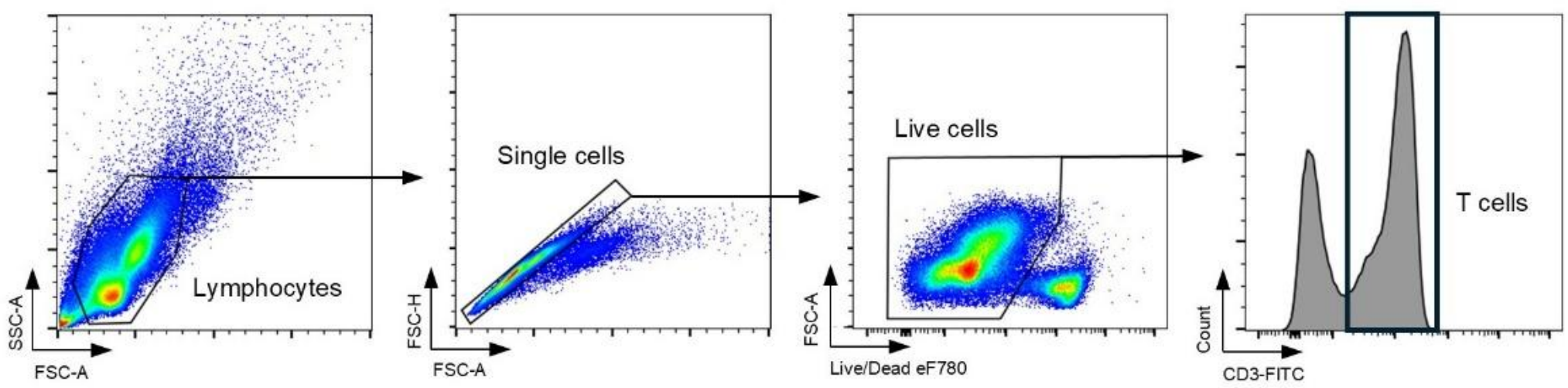

Supplementary Figure 2. Gating strategy used to identify T cells in PBMCs. Lymphocytes were first identified by their light scatter properties, followed by singlet gating and exclusion of dead cells. Within the live cell population, T cells were identified by their CD3 expression.

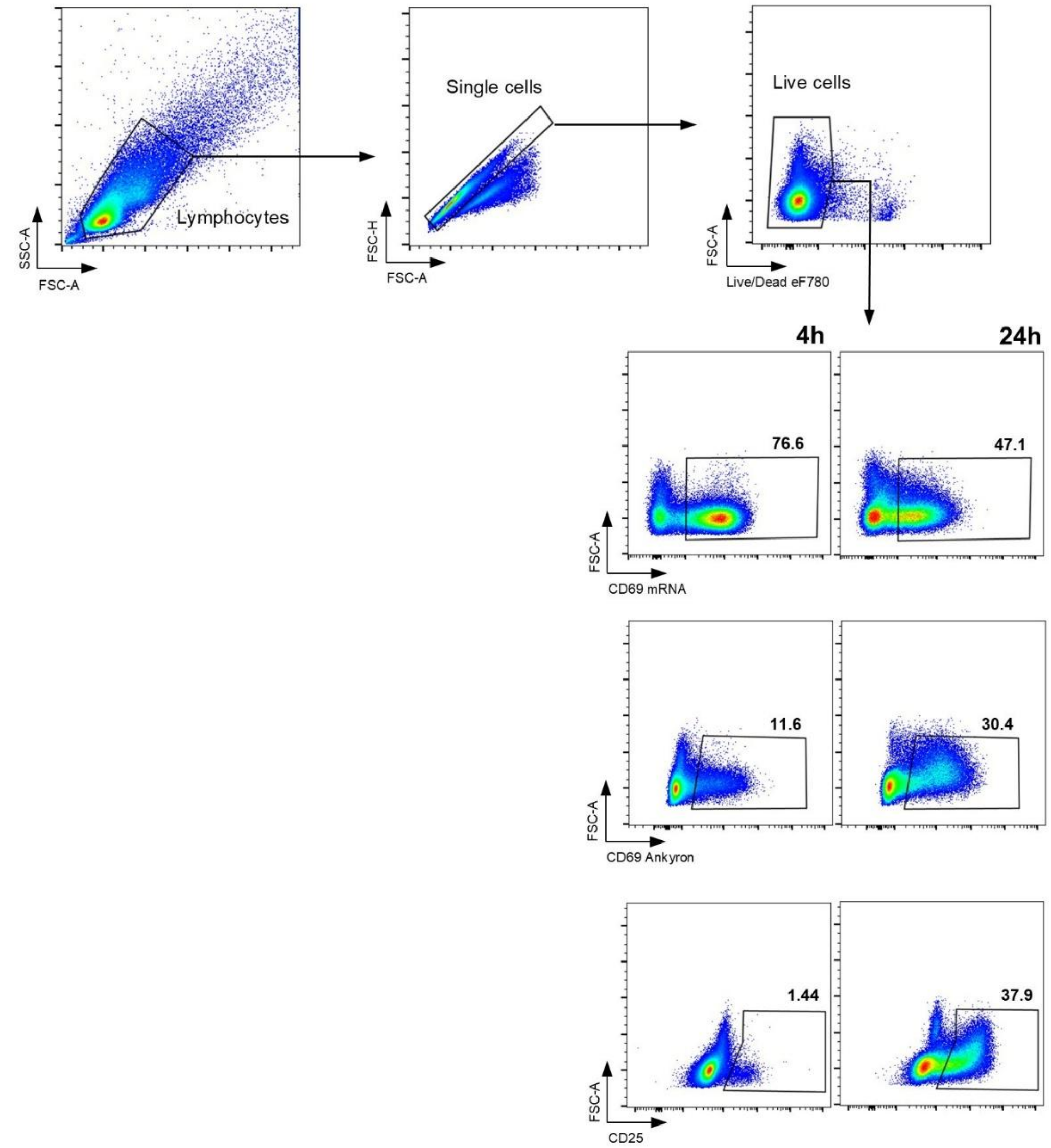

Supplementary Figure 3. Gating strategy used to analyze CD69 mRNA, CD69 protein and CD25 protein expression within live PBMCs over time. Lymphocytes were first identified by their light scatter properties, followed by singlet gating and exclusion of dead cells. Within the live cell population, expression of CD69 mRNA (PrimeFlow^{TM} RNA Assay), CD69 protein (Ankyron^{+}) or CD25 protein was analyzed. Flow cytometry plots show representative gating 4h and 24h after PMA/ionomycin stimulation.

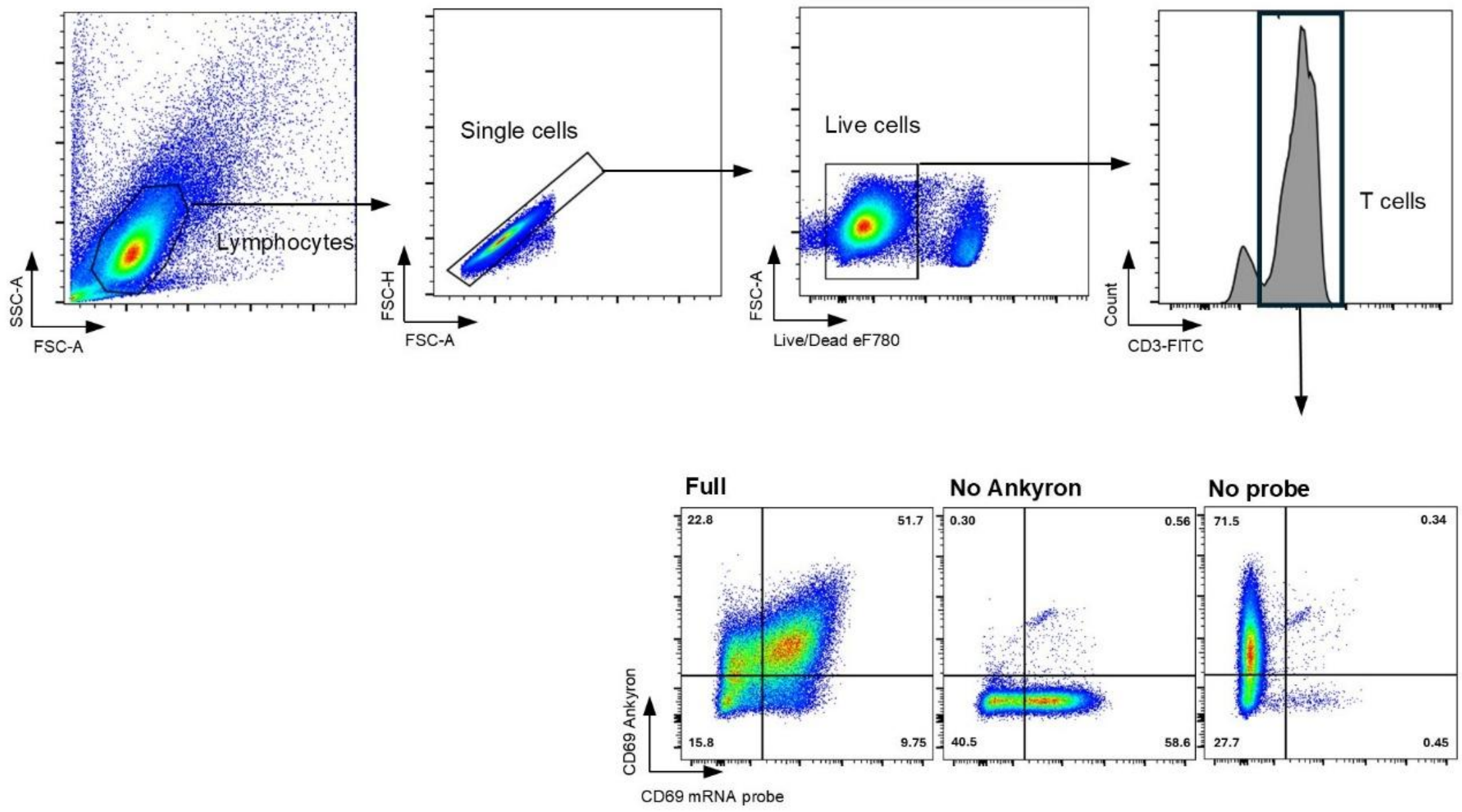

Supplementary Figure 4. Gating strategy of CD69 Ankyron/mRNA positive T cells (PrimeFlow™ RNA Assay). Lymphocytes were first identified by their light scatter properties, followed by singlet gating and exclusion of dead cells. Within the live cell population, T cells were identified by their CD3 expression. Frequencies of CD69 Ankyron^{+} and CD69 mRNA^{+} cells within live T cells were determined. FMO controls for the CD69 Ankyron and the CD69 mRNA label probe are shown for PMA/ionomycin stimulated cells.

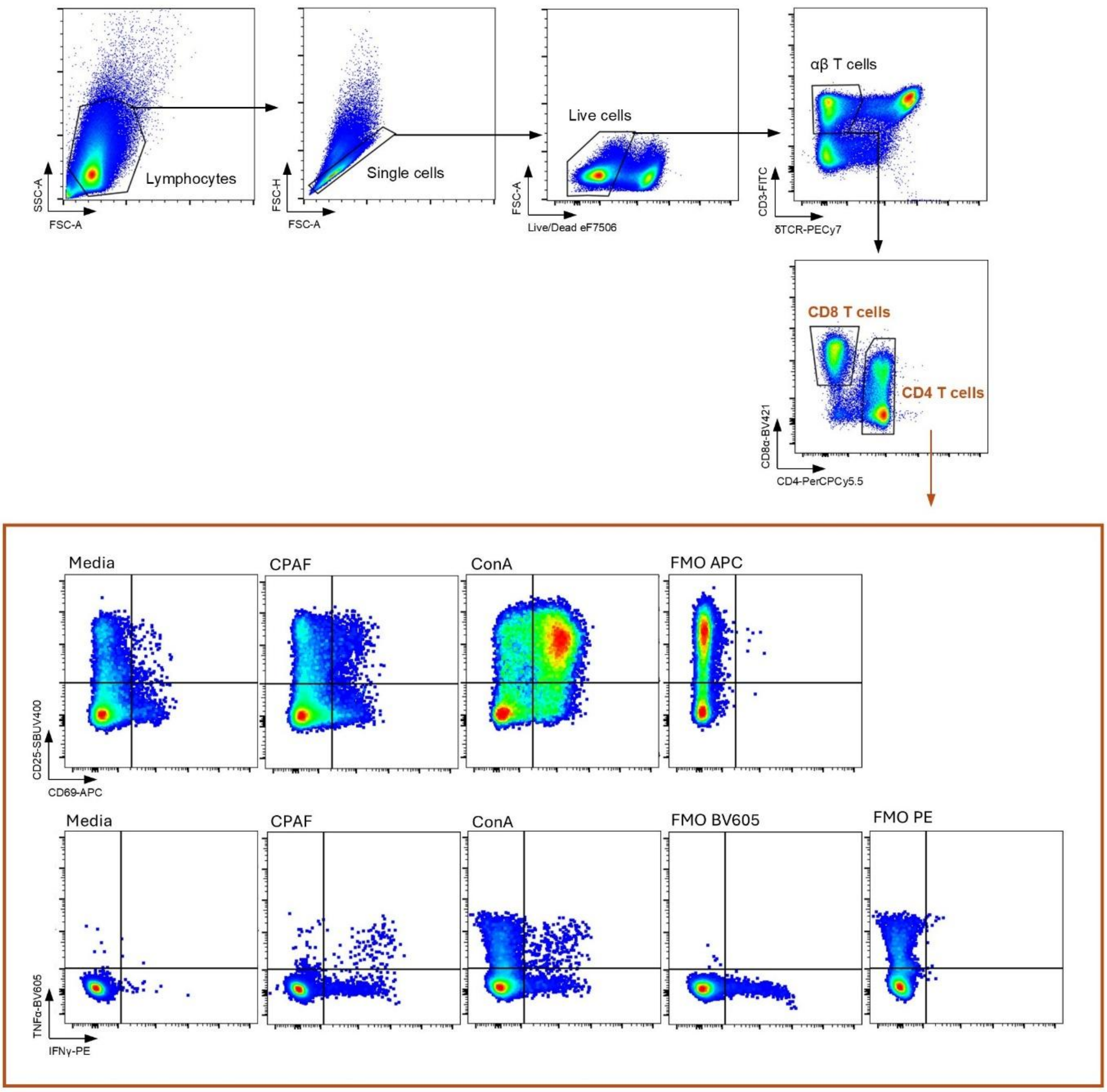

Supplementary Figure 5. Gating strategy for the evaluation of antigen-specific expression of CD69 and CD25. Lymphocytes were first identified by their light scatter properties, followed by singlet gating and exclusion of dead cells. Within the live cell population, γδ T cells were defined as TCR-γδ^{+} and αβ T cells as CD3^{+}TCR-γδ^{-}. Alpha-beta T cells were further subdivided into CD4+ and CD8α+ T cells and assessed for their expression of CD25 and CD69, as well as intracellular cytokine production (IFNγ , TNFα) under different stimulation conditions (media = negative control, CPAF = vaccine antigen, ConA = positive control). FMO = fluorescence minus one control.

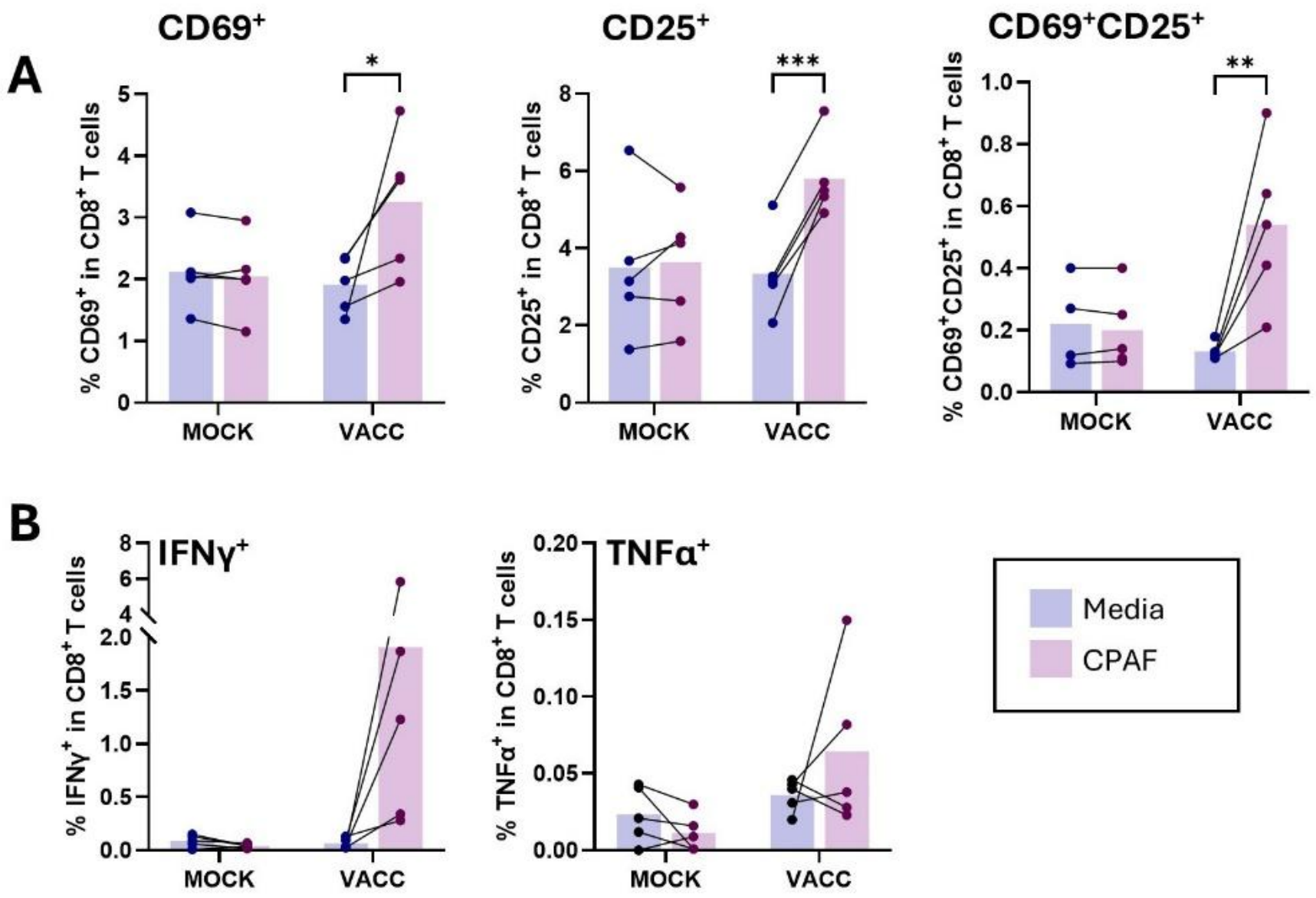

Supplementary Figure 6. In vitro stimulation with vaccine antigen (CPAF) leads to upregulation of CD69/CD25 on CD8 T cells from vaccinated pigs. Pigs were either vaccinated with CPAF adjuvanted with TriAdj (VACC) or received TriAdj adjuvant alone (MOCK) as outlined in **Figure 5A**. Cryopreserved PBMCs from day 13 post-vaccination were thawed and stimulated with an overlapping CPAF peptide pool or left unstimulated (media control) for 18 hours. Following gating on live and singlet cells, CD8 T cells were identified and analyzed for activation marker and cytokine expression as shown in **Supplementary Figure 5**. **(A)** shows the expression of CD69 and CD25 on CD8 T cells from MOCK and VACC groups, with or without CPAF stimulation. **(B)** Frequencies of IFNγ^{+} or TNFα^{+} CD8 T cells in unstimulated and CPAF-stimulated samples. Each dot in the bar graphs represents one individual pig (n = 5 per group). The statistical analysis was performed using GraphPad Prism, applying two-way RM ANOVA with Bonferroni multiple comparisons test. The statistical analysis of within-group comparisons is shown. * $p \leq 0.05$, ** $p \leq 0.01$, *** $p \leq 0.001$.

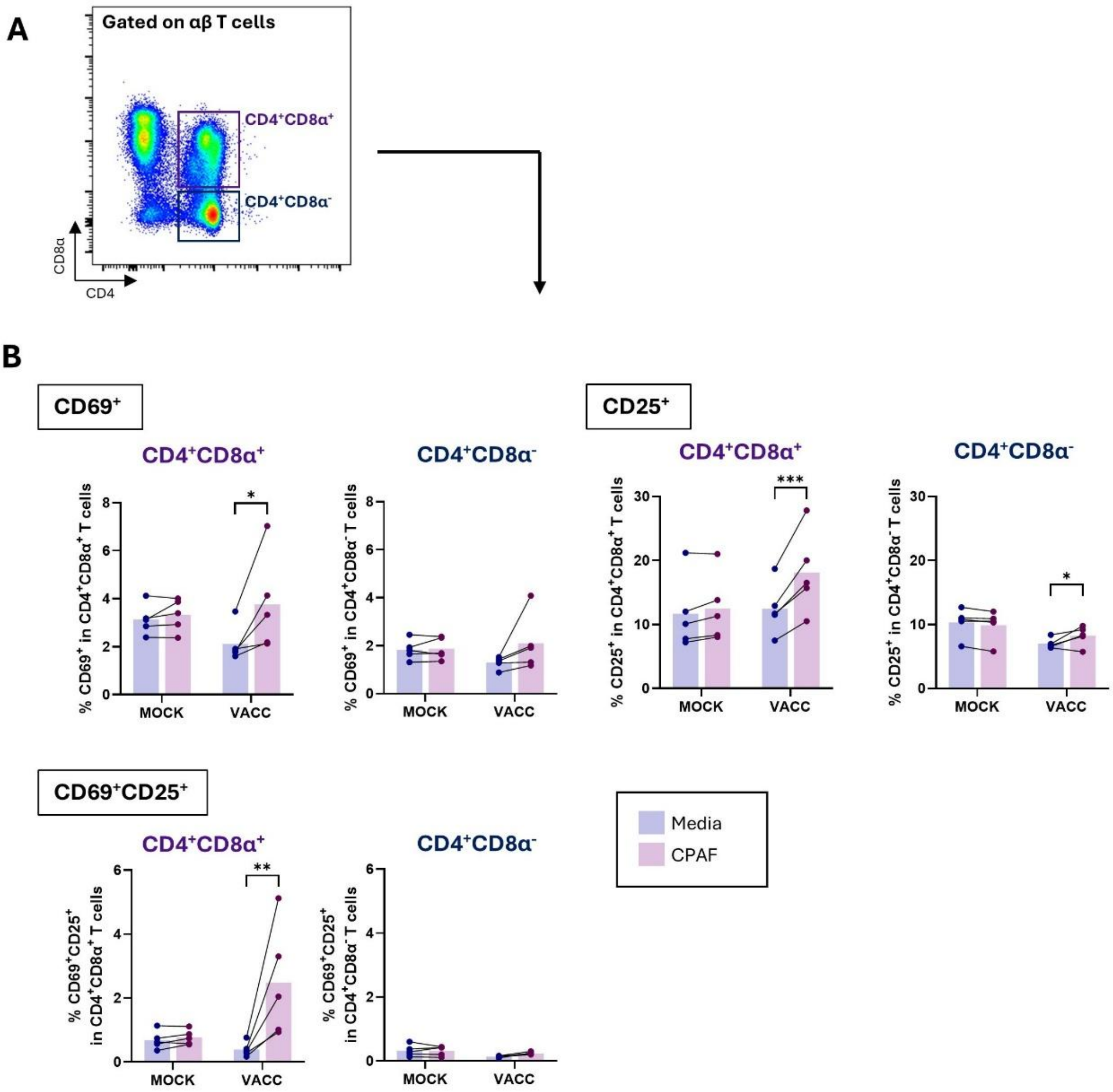

Supplementary Figure 7. Antigen-specific upregulation of CD69/CD25 on CD4 T cells from vaccinated pigs is mainly driven by the $CD4^+CD8\alpha^+$ subset. Pigs were either vaccinated with CPAF adjuvanted with TriAdj (VACC) or received TriAdj adjuvant alone (MOCK) as outlined in **Figure 5A**. Cryopreserved PBMCs from day 13 post-vaccination were thawed and stimulated with an overlapping CPAF peptide pool or left unstimulated (media control) for 20 hours. Following gating on live and singlet cells, αβ T cells ($CD3^+$ δTCR^-) were identified and the $CD4^+CD8\alpha^+$ and $CD4^+CD8\alpha^-$ subsets were analyzed for the expression of activation markers as shown in **Supplementary Figure 5 and (A)**. **(B)** shows the expression of CD69 and CD25 within CD8α defined CD4 T cells from MOCK and VACC groups, with or without CPAF stimulation. Each dot in the bar graphs represents one individual pig (n = 5 per group). The statistical analysis was performed using GraphPad Prism, applying two-way RM ANOVA with Bonferroni multiple comparisons test. The statistical analysis of within-group comparisons is shown. * $p \leq 0.05$, ** $p \leq 0.01$, *** $p \leq 0.001$.

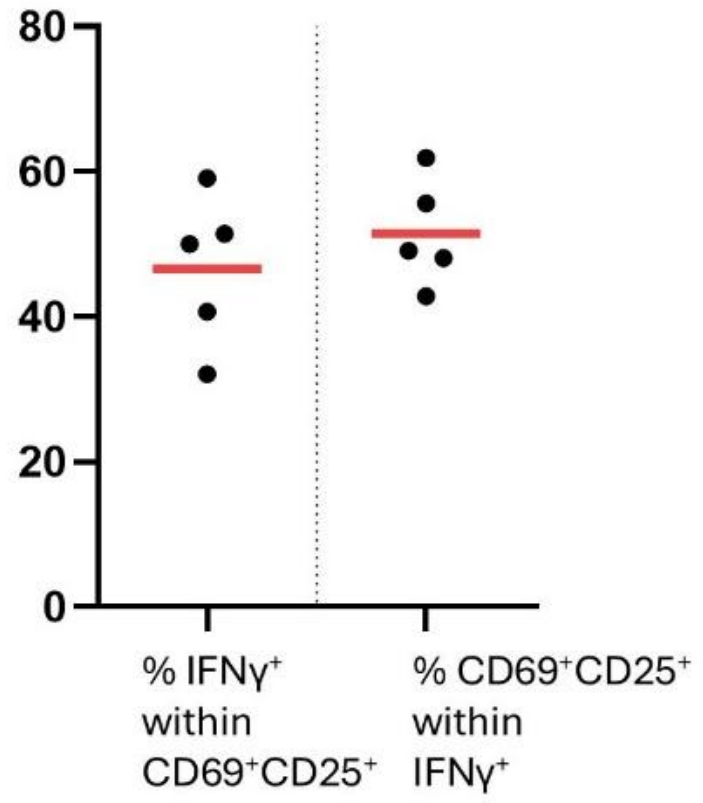

Supplementary Figure 8. Overlap of CD69/CD25 phenotype and IFNγ production within CD4 T cells from vaccinated pigs. Pigs were vaccinated with CPAF adjuvanted with TriAdj (VACC) as outlined in **Figure 6A**. Cryopreserved PBMCs from day 13 post-vaccination were thawed and stimulated with an overlapping CPAF peptide pool for 18 hours. Following gating on live and singlet cells, CD4 T cells were identified and analyzed for activation marker and cytokine expression. The bar graph shows the proportion of IFNγ$^+$ cells among CD69$^+$CD25$^+$ CD4 T cells (left) and the proportion of CD69$^+$CD25$^+$ cells among IFNγ$^+$ CD4 T cells. Each dot in the bar graphs represents one individual pig (n = 5 per group).
